# Periostin Promotes Neural Stem Cell Proliferation and Differentiation following Hypoxic-Ischemic Injury

**DOI:** 10.1371/journal.pone.0123585

**Published:** 2015-04-20

**Authors:** Si-Min Ma, Long-Xia Chen, Yi-Feng Lin, Hu Yan, Jing-Wen Lv, Man Xiong, Jin Li, Guo-Qiang Cheng, Yi Yang, Zi-Long Qiu, Wen-Hao Zhou

**Affiliations:** 1 Department of Neonatology, Children’s Hospital of Fudan University, Shanghai, China; 2 Key Laboratory of Birth Defect, Children’s Hospital of Fudan University, Shanghai, China; 3 Key Laboratory of Neonatal Diseases, Ministry of Health, Children’s Hospital of Fudan University, Shanghai, China; 4 Institute of Neuroscience, Shanghai Institutes for Biological Sciences, Chinese Academy of Sciences, Shanghai, China; 5 Institutes of Biomedical Sciences, Fudan University, Shanghai, China; University of Cincinnati, College of Medicine, UNITED STATES

## Abstract

Neural stem cell (NSC) proliferation and differentiation are required to replace neurons damaged or lost after hypoxic-ischemic events and recover brain function. Periostin (POSTN), a novel matricellular protein, plays pivotal roles in the survival, migration, and regeneration of various cell types, but its function in NSCs of neonatal rodent brain is still unknown. The purpose of this study was to investigate the role of POSTN in NSCs following hypoxia-ischemia (HI). We found that POSTN mRNA levels significantly increased in differentiating NSCs. The proliferation and differentiation of NSCs in the hippocampus is compromised in POSTN knockout mice. Moreover, NSC proliferation and differentiation into neurons and astrocytes significantly increased in cultured NSCs treated with recombinant POSTN. Consistently, injection of POSTN into neonatal hypoxic-ischemic rat brains stimulated NSC proliferation and differentiation in the subventricular and subgranular zones after 7 and 14 days of brain injury. Lastly, POSTN treatment significantly improved the spatial learning deficits of rats subjected to HI. These results suggest that POSTN significantly enhances NSC proliferation and differentiation after HI, and provides new insights into therapeutic strategies for the treatment of hypoxic-ischemic encephalopathy.

## Introduction

Hypoxic-ischemia (HI) encephalopathy in neonates is a problem of enormous public health importance [1, 2]. Most of the survivors have neurodevelopmental sequela, including cerebral palsy and cognitive/behavioural/attentional deficits, especially in developing countries [1, 2]. There are few clinically successful strategies for neuroprotection once damage has occurred [3, 4].

Neurogenesis occurs in two regions of the adult rodent brain, the subventricular zone (SVZ) and the subgranular zone (SGZ) of the hippocampus dentate gyrus [5]. The persistence of neurogenesis throughout the lifetime raises the possibility that resident neural stem cells (NSCs) can mount a regenerative response to replace neurons that are lost upon brain injury. NSCs give rise to the three main cerebral cell types, which are neurons, astrocytes, and oligodendrocytes [6]. Previous studies have shown that endogenous neurogenesis increases in the immature rat brain in response to hypoxic-ischemic insult [7–9], which may serve to rescue neuronal loss and restore cognitive function after brain injury [10].

Periostin (POSTN; also PN or OSF-2) is a secreted extracellular matrix glycoprotein with a role in cell adhesion [11, 12]. POSTN is transiently expressed in multiple tissues, including the CNS, during early development. In the mouse CNS, POSTN expression spans from embryonic day 12.5 (E12.5) to 19.5 (E19.5) [13], a period that coincides with a critical period for neuron maturation and axon extension. Increased expression of POSTN after injury has been found in several adult tissues, such as fractured-bone-infarcted myocardial tissue and tissue undergoing cutaneous wound healing [14–16]. Delivery of recombinant POSTN has also been shown to promote cardiomyocyte proliferation and to improve cardiac remodeling [17, 18]. These data suggest an important role for POSTN in tissue repair and regeneration. Moreover, POSTN contains four domains that share homology with the insect growth cone guidance protein fasciclin I, suggesting a role for POSTN in neurite outgrowth [19]. Recently, POSTN has been shown to be re-expressed in the CNS after stroke and to reduce infarct volume when administered via intracerebroventricular injection [20]. From the above viewpoint, we hypothesize that POSTN may play an important role in brain repair and function recovery after hypoxic-ischemic injury.

Therefore, the purpose of the present study was to test the influence of POSTN deficiency on NSC proliferation and differentiation into neurons, and to examine the role(s) of POSTN in cultured NSCs and in neonatal rat brain after hypoxic-ischemic injury. The results indicate that POSTN is robustly expressed in differentiating NSCs, and stimulates NSC proliferation and differentiation *in vitro* and *in vivo*. POSTN treatment also rescued the cognitive deficits associated with hypoxia-ischemia (HI). These findings reveal novel functions of POSTN in the developing rodent brain, and offer a potential avenue for therapeutic intervention after HI-induced brain injury.

## Materials and Methods

### Animal surgery and HI induction

This study was conducted in accordance with the National Institutes of Health Guide for the Care and Use of Laboratory Animals. All protocols were approved by Clinical Pharmacology Ethics Committee of Fudan University, Pediatrics Ethics Committee in Shanghai. *POSTN* ^*+/-*^ mice were purchased from Jackson Laboratory (Stock No.009067; B6:129-POSTN^tm1Jmol/^J; Heterozygous X Heterozygous). We interbred mice that were heterozygous carriers of this mutation and obtained wild-type (*POSTN* ^*+/+*^) and homozygous mutant (*POSTN* ^*-/-*^) offspring with the expected Mendelian distribution. DNA analyzed by PCR was used to identify POSTN heterozygous (*POSTN* ^*+/-*^ *)*, homozygous mutant (*POSTN* ^*-/-*^) and wild-type (*POSTN* ^*+/+*^)mice. The pups were nursed by their biological mothers until they were sacrificed. The pups were given intraperitoneal injection with freshly prepared 5-bromodeoxyuridine (BrdU; Sigma, 50 mg/kg body weight) once daily until the time of sacrifice [21].

HI was induced in neonatal Sprague-Dawley rats at postnatal day 7 (P7) as previously described [22–24]. Briefly, male or female pups were anesthetized with isoflurane, and the left common carotid artery was isolated and ligated with surgical silk. The surgical procedure was completed within 5 min and the pups were allowed to recover for 2 h in a temperature-controlled incubator. Animals were then placed in a container perfused with a humidified gas mixture (8% oxygen-nitrogen) at 37°C for 2 h. Control animals were not subjected to the surgical procedure. The animals were randomly assigned to four groups, where control and HI-subjected pups were injected with either PBS or POSTN following the recovery period (Sham + PBS, Sham + POSTN, HI + PBS, and HI + POSTN). Specifically, 2 μl PBS or recombinant human POSTN (100 μg/ml; Abcam, UK) was injected into the lateral ventricles as previously described [25]. The pups were nursed by their biological mothers until they were sacrificed at 7 or 14 days after HI. To label proliferating cells, the pups were given intraperitoneal injection with freshly prepared 5-bromodeoxyuridine (BrdU; 50 mg/kg body weight) once daily after HI until the time of sacrifice [21].

### Tissue preparation and immunofluorescence

Both wild-type (*POSTN* ^*+/+*^) and homozygous mutant (*POSTN* ^*-/-*^) mice were deeply anesthetized on P7, 14, 21, and 28; and SD rat pups from control and experimental groups were deeply anesthetized at 7 and 14 days after HI, and perfused with 4% paraformaldehyde in 0.1M PBS. The brains were removed and immersion-fixed in the same solution at 4°C for 24 h, then dehydrated in a graded series of sucrose (20%, 30% in PBS) until they were saturated. Coronal sections were cut on a freezing microtome (Jung Histocut Model 820-II; Leica, Nussloch, Germany) at a thickness of 30 μm from Bregma 1.60 to −4.80 mm, and stored at −20°C in cryoprotectant solution. For combined immunodetection of BrdU and cell type-specific markers in NSCs, sections were first incubated with anti-BrdU antibody (1:250; Santa Cruz, Sc-32323, USA) for 2 h at 37°C, then overnight at 4°C. Sections were rinsed with PBS, then incubated with anti-mouse IgG-Cy3 (1:500; Invitrogen, USA) for 45 min at 37°C, followed by an overnight incubation with anti-nestin (1:100; Millipore, MAB353, USA), anti-NeuN (1:200; Millipore, MAB377, USA), anti-MAP2 (1:200; MAB5622, Millipore, USA), or anti-GFAP (1:150; AB5804, Millipore, USA) at 4°C. After rinsing in PBS, sections were incubated with anti-rabbit IgG-FITC (1:500; Invitrogen) or anti-sheep IgG-FITC (1:500; Invitrogen) for 45 min at 37°C. Sections were washed and mounted onto glass slides using fluorescence mounting medium (Vector Laboratories, USA). Fluorescence was detected with excitation/emission wavelengths of 570/576 nm (Rhodamine) and 490/525 nm (FITC) using a Zeiss 510 confocal laser scanning microscope (Zeiss, Thornwood, USA).

### Neural Stem Cell culture

NSCs were obtained from dissociated embryonic cortex dissected from C57BL/6 mice at E12.5 [26]. The tissue was triturated and filtered before centrifugation, and cell suspensions were plated at a density of 2 × 10^6^ cells/ml with DMEM/F12 medium containing 1% N2, 2% B27 (Gibco, Carlsbad, USA), EGF (20 ng/ml; R&D Systems), and bFGF (20 ng/ml; R&D Systems). Differentiation was induced by replacing the culture medium with a DMEM/F12 medium containing 1% fetal bovine serum (Gibco) in addition to 1% N2 and 2% B27, but lacking growth factors. The cells were treated with BrdU at 3, 5, and 7 days after switching to differentiation medium (growth factor withdrawal).

### POSTN mRNA quantification by real-time PCR

500 ng of total RNA was extracted from the NSCs at 0 h, 48 h, and 5 days after differentiation using TRIZOL. The RNA was reverse-transcribed using PrimeScript RT Master Mix (Takara Bio Inc., Shiga, Japan) according to the manufacturer’s instructions. 1 μl of cDNA was used in 20 μl reactions that consisted of 1× SYBR Green Realtime PCR Master Mix (Toyobo, Osaka, Japan), 0.5 μM forward and reverse primers, and nuclease-free water. GAPDH expression levels were determined in parallel as an internal control for all the samples. PCR reactions were performed using Qiagen Rotor-Gene Q (Qiagen, USA). Primers for POSTN and GAPDH mRNA detection were designed with PrimerBank. The sequences were as follows: for POSTN, 5’-GGGAGCCACTACCACTCAG-3’ (forward) and 5’-GTACGTGTAT GACCCTTTTCCTT-3’ (reverse); and for GAPDH, 5’-ACGGCCGCATCTTCTTGT GCAGTG-3’ (forward) and 5’-GGCCTTGACTGTGCCGTTGAATTT-3’ (reverse). All reactions were performed in triplicate, and relative quantification values were calculated using the ΔCt method (95% CI) with calibration to 0 h samples. All primer sets were subjected to a dissociation curve analysis and produced single peaks on a derivative plot of raw fluorescence.

### Morris water maze test

Animals subjected to the HI surgical procedure described above were tested for cognitive performance in the Morris water maze at P42–46 (n = 20 animals per group). The water maze (Med Associates Inc., Georgia, USA) (diameter: 1.6 m; height: 0.5 m; distance from floor to bottom of maze: 0.45 m) was filled with water (21 ± 1°C). The black platform (diameter: 0.12 m; height: 0.30 m) was submerged to 1 cm below the water surface. The memory tests were performed as previously described [27]. For the navigation test, the animals underwent four trials daily with different starting points for four consecutive days. Animals escaping the platform were restricted within 120 s, and were allowed to rest on the platform for 15 s. After removal of the platform on the fifth day, animals were subjected to a 60 s space probe trial. The escape latency, swimming distance, number of platform crossings, and the percentage of time spent in the target quadrant were recorded. Data were analyzed using a tracking program (DigBehv-MWM; Shanghai Jiliang Software Technology Co. Ltd., Shanghai, China).

### Statistical methods

Three consecutive sections were selected for the quantification of NSCs in immunostained tissue. Double-labeled cells in the SVZ and SGZ were counted using Stereo Investigator 6.5 software (MicroBrightfield Inc., Williston, USA) using the Optical Fractionator probe at 20× objective (n = 8–10 animals per group) [28]. For cultured NSCs, six images per treatment condition from randomly selected regions of the coverslip were acquired using a 40× objective lens, and double-labeled cells were counted manually. Data from three independent experiments were pooled and expressed as mean ± SEM. Differences between groups were analyzed by ANOVA followed by the Student-Newman-Keuls test. Statistical significance was set at *P* < 0.05.

## Results

### POSTN expression was increased when accompanied by NSC differentiation

**Fig 1:**
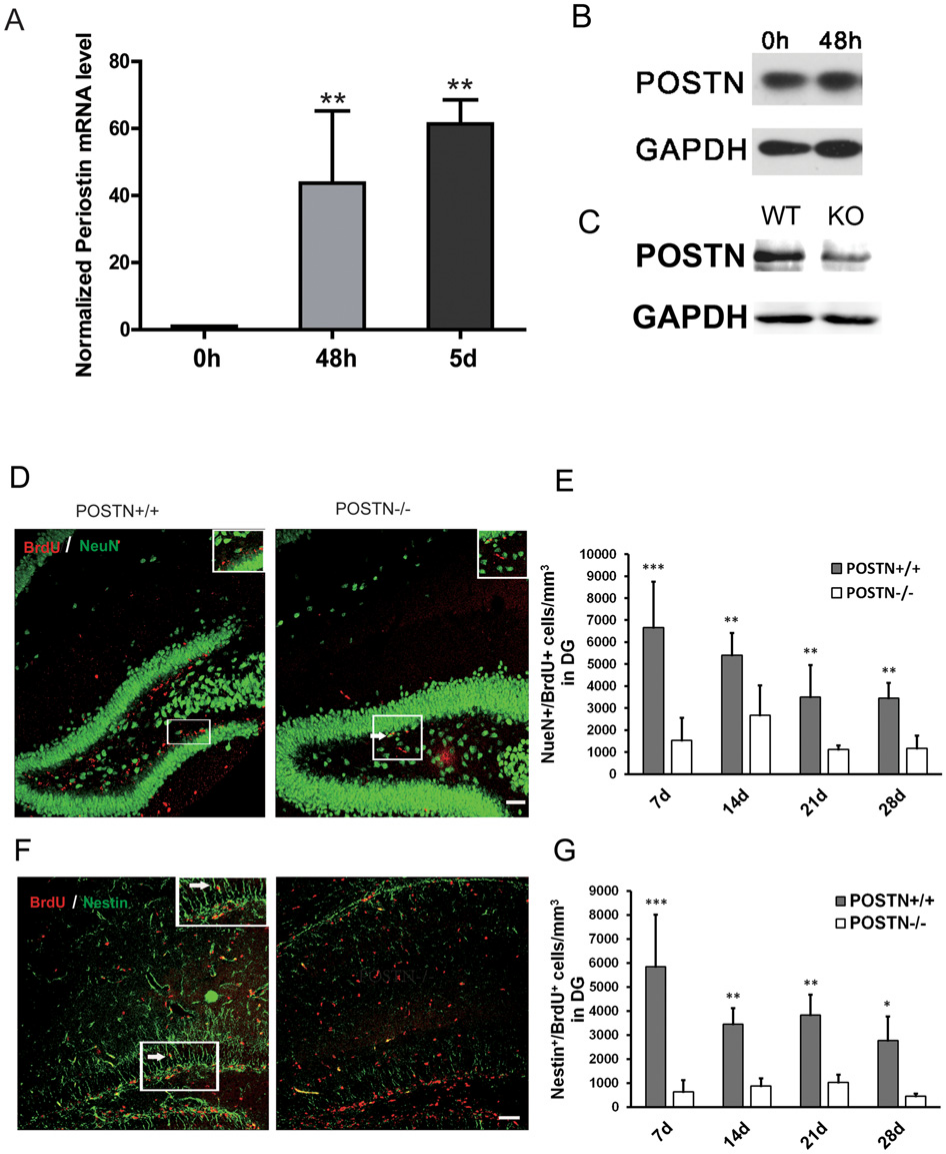
Expression of POSTN mRNA in cultured NSCs and the effect of POSTN deficiency on NSC proliferation and differentiation in SGZ. A: Compared to POSTN expression in proliferating NSCs (0 h), POSTN levels were increased at 48 h and 5 days following differentiation. B: POSTN is stably expressed in mouse neural stem cells. C: The protein expression of POSTN was decreased in the brain of *POSTN* ^*+/-*^ mice (KO) compared to wild-type mice (WT). D-E: The number of proliferating neurons was decreased at P7, 14, 21 and 28 in POSTN null mice. F-G: The number of proliferating NSCs was decreased in POSTN null mice at P7, 14, 21 and 28. Cells were quantified per mm^3^ and data are shown as mean ± SEM. * *P* < 0.05; ** *P* < 0.01; n = 9 animals per group. Scale bar = 50μm.

POSTN expression in mouse CNS spans from E12.5 to E19.5 [13]. To investigate the manner of POSTN expression during NSC differentiation, NSCs isolated from E12.5 mice were cultured in proliferation and differentiation media successively. The levels of POSTN gene expression at 0 h, 48 h and 5 days after differentiation were determined by quantitative real-time PCR. POSTN mRNA levels were significantly increased at 48 h and 5 days following NSC differentiation compared to proliferating NSCs (0 h) (Fig 1A). We also detected the POSTN protein expression in mouse NSCs. Western blot analysis showed that POSTN was stably expressed in mouse NSCs (Fig 1B).

### Defects in proliferation and Differentiation of NSCs in *POSTN* null mouse

*POSTN* ^*+/-*^ mice were purchased from Jackson Laboratory. The absence of protein expression was comfirmed by western blot analysis on P7 developing brain extracts (Fig 1C). To examine the influence of POSTN deficiency on proliferation and differentiation of neural stem cells, *POSTN* ^*+/-*^ mice were intercrossed to obtain homozygous *POSTN* ^*-/-*^ mice. BrdU and Nestin double-immunostaining was used to label the proliferating NSCs. BrdU and NeuN double-immunostaining was used to label new mature proliferating neurons. Compared to the *POSTN* ^*+/+*^ mice, both proliferating mature neurons (Fig 1D and 1E) and neural stem cells (Fig 1F and 1G) in the dentate gyrus (DG) of the hippocampus were significantly decreased in the *POSTN* ^*-/-*^ mice at P7, 14, 21 and 28.

### POSTN promotes NSC proliferation and differentiation *in vitro*

**Fig 2:**
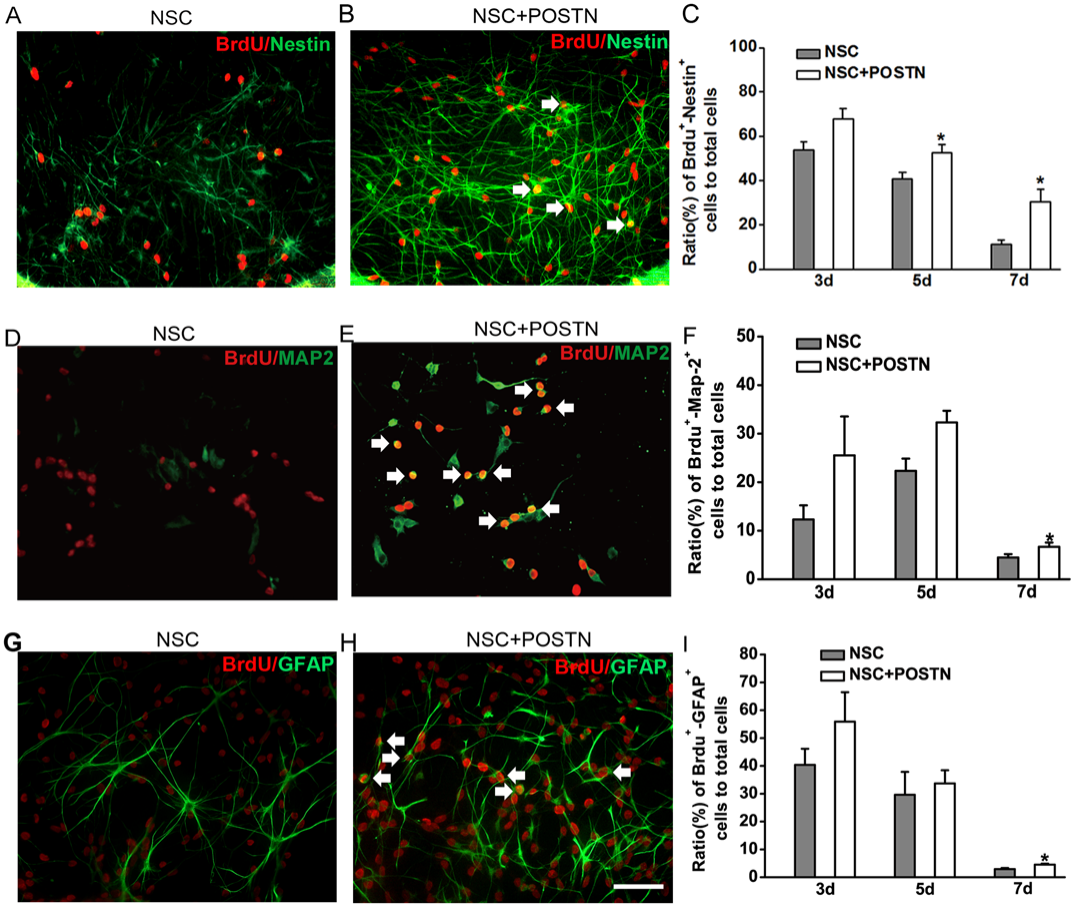
Effects of POSTN on NSC proliferation and differentiation *in vitro*. A–C: The presence of POSTN (+POSTN) increases the number of proliferating NSCs at 5 and 7 days after plating, as evidenced by an increase in BrdU^+^/Nestin^+^ double-positive cells. D–F: The fraction of NSCs undergoing differentiation into neurons (BrdU^+^/MAP2^+^ cells) increases at 7 days after plating when POSTN is present. G–I: The fraction of NSCs undergoing differentiation into astrocytes (BrdU^+^/GFAP^+^ cells) increased at 7 days after plating in the presence of POSTN. The ratio of double-positive cells to the total number of cells is shown as mean ± SEM (* *P* < 0.05 from three independent experiments). Scale bar = 100 μm.

To investigate the effects of POSTN on NSC proliferation and differentiation *in vitro*, NSCs were cultured in the presence of 50 ng/mL POSTN. Proliferating NSCs were identified as cells double-positive for BrdU and Nestin (BrdU^+^/Nestin^+^), while BrdU^+^/MAP2^+^ and BrdU^+^/GFAP^+^ cells represented NSCs differentiating into neurons and astrocytes, respectively. NSCs cultured in the presence of POSTN showed a prominent enhancement in proliferation (Fig 2A and 2B). Proliferation rates were significantly increased at 5 and 7 days after NSC differentiation compared to control NSCs (Fig 2C). Similarly, the numbers of neurons (Fig 2D–2F) and astrocytes (Fig 2G–2I) were increased after POSTN treatment compared to controls, although these increases were not statistically significant until 7 days after differentiation.

### POSTN promotes NSC proliferation and differentiation in the SVZ and SGZ in the neonatal brain after HI

**Fig 3:**
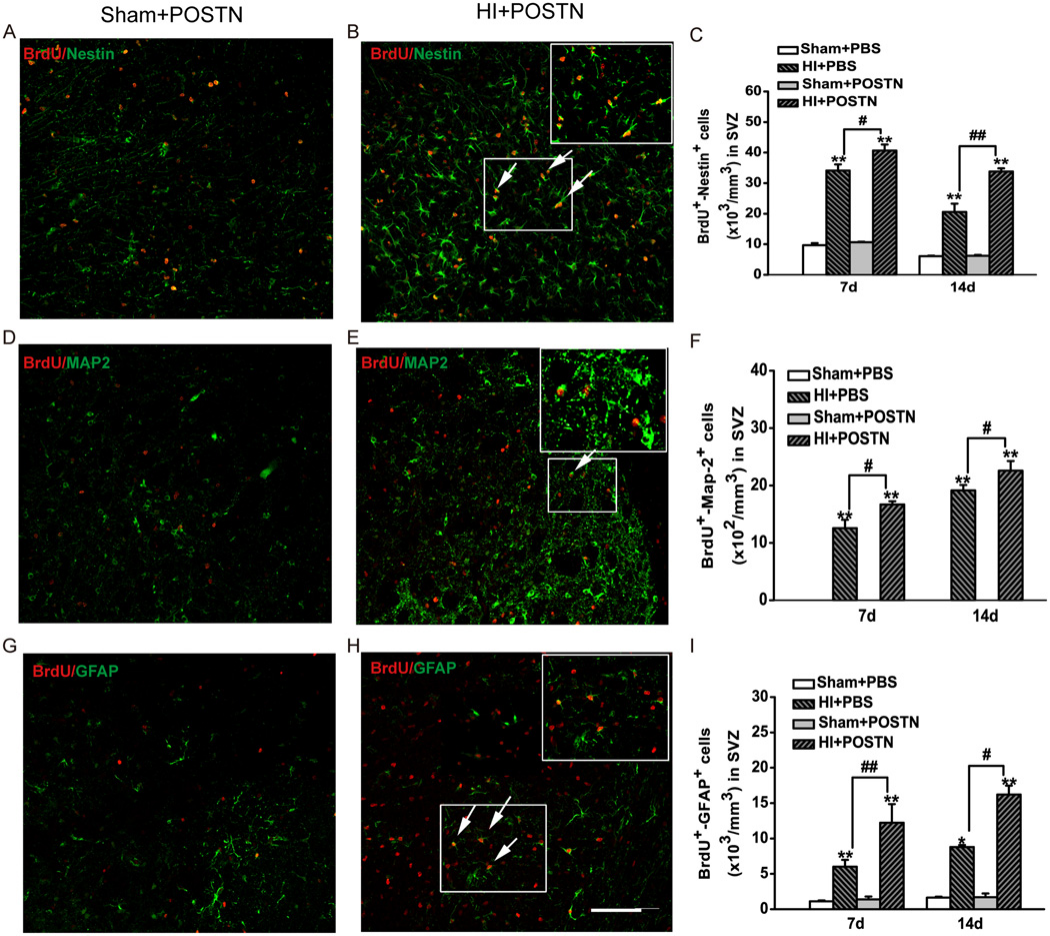
Effects of POSTN on NSC proliferation and differentiation in the rat SVZ following HI. Hypoxic-ischemic injury (HI + PBS) stimulates NSC proliferation (A–C; BrdU^+^/Nestin^+^ cells) and differentiation into neurons (D–F; BrdU^+^/MAP2^+^ cells) and astrocytes (G–I; BrdU^+^/GFAP^+^ cells) at 7 and 14 days, but the effect is potentiated when POSTN is present (HI + POSTN). Cells were quantified per mm^3^ and data are shown as mean ± SEM. * or # *P* < 0.05; ** or ## *P* < 0.01; n = 8 animals per group. Scale bar = 100 μm.

**Fig 4:**
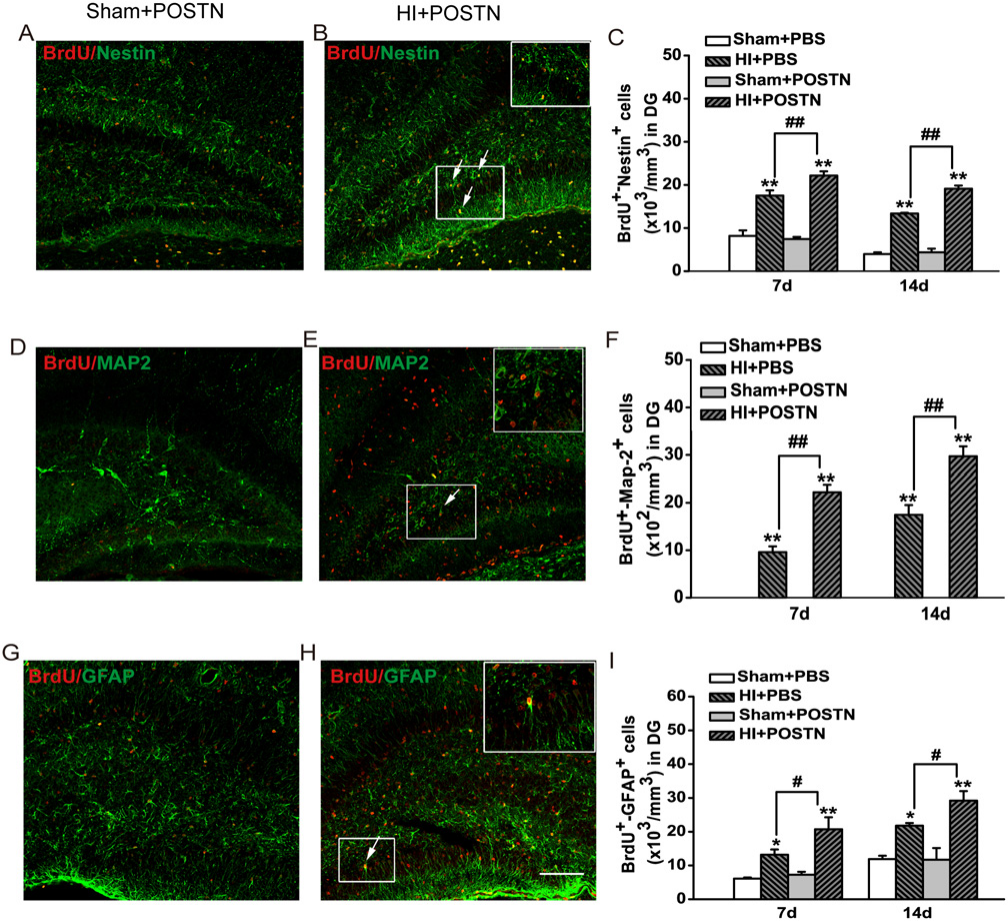
Effects of POSTN on NSC proliferation and differentiation in the SGZ following HI. Hypoxic-ischemic injury (HI + PBS) stimulates NSC proliferation (A–C; BrdU^+^/Nestin^+^ cells) and differentiation into neurons (D–F; BrdU^+^/MAP2^+^ cells) and astrocytes (G–I; BrdU^+^/GFAP^+^ cells) at 7 and 14 days, but the effect is potentiated when POSTN is present (HI + POSTN). Cells were quantified per mm^3^ and data are shown as mean ± SEM. * or # *P* < 0.05; ** or ## *P* < 0.01; n = 8 animals per group. Scale bar = 100 μm.

Previous studies found that POSTN expression was increased in different tissues after injury [14–16]. Also, POSTN exhibited neuroprotective function in a transient middle cerebral artery occlusion model [20]. To examine the effects of POSTN on NSC proliferation and differentiation *in vivo*, recombinant POSTN was injected into the lateral ventricles of neonates following HI injury. Proliferating and differentiating NSCs were identified by combined immunodetection of BrdU and either Nestin, MAP2, or GFAP in the SVZ and SGZ. HI injury caused significant increases in the proliferation and differentiation of NSCs (HI+PBS) compared to animals that were not subjected to hypoxic-ischemic conditions (Sham+ PBS) (Fig 3 and 4). However, the number of BrdU^+^/Nestin^+^ cells in the SVZ was markedly increased after POSTN treatment (HI + POSTN) at 7 and 14 days (Fig 3A–3C). In contrast, treatment of POSTN in the absence of HI injury (Sham +POSTN) had no effect on NSC proliferation. Similar increases in BrdU^+^/MAP2^+^ (Fig 3D–3F) and BrdU^+^/GFAP^+^ (Fig 3G–3I) double-positive cells were observed after POSTN treatment, reflecting an increase in differentiation of NSCs into neurons and astrocytes, respectively. Consistent observations were also made for NSCs in the SGZ (Fig 4). The number of BrdU^+^/Nestin^+^ cells in the HI + POSTN group was significantly higher than those in HI + PBS-treated animals (Fig 4A–4C), demonstrating that NSC proliferation was stimulated by POSTN. The numbers of differentiated neurons (BrdU^+^/MAP2^+^ cells; Fig 4D–4F) and astrocytes (BrdU^+^/GFAP^+^ cells; Fig 4G–4I) were also higher in the HI + POSTN group at 7 and 14 days after HI compared to controls, consistent with a general effect of promoting differentiation rather than of specifying a particular cell type.

### Cognitive enhancement following POSTN treatment

**Fig 5:**
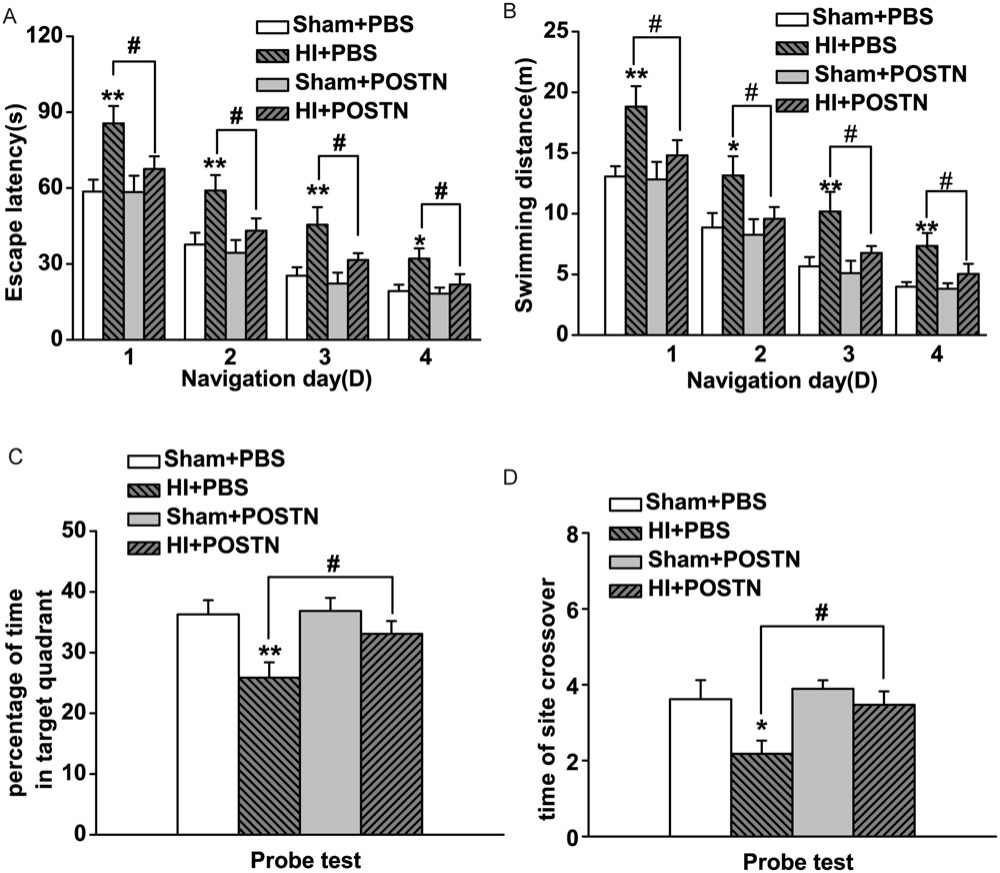
Effect of POSTN on promoting cognitive deficits after HI. Perinatal POSTN injection (HI + POSTN) rescues cognitive deficits caused by HI (HI + PBS) at P42–46, as assessed by escape latency (A), swimming distance (B), percentage of time spent in the target quadrant (C), and number of platform site crossovers (D) in the Morris water maze test. Data are shown as mean ± SEM. * or # *P* < 0.05; ** *P* < 0.01; n = 20 animals per group.

To examine the effect of POSTN on cognitive function, which is impaired by hypoxic-ischemic injury, animals were tested in the Morris water maze at P42–46. In the navigation trials, it was observed that HI + PBS-treated control animals had greater escape latency (Fig 5A) and swimming distance (Fig 5B) compared to animals that had not undergone HI surgery, indicating a learning deficit resulting from HI. However, HI + POSTN-treated animals showed significant improvement in escape latency and swimming distance, which increased by 20% and 30%, respectively (Fig 5A and 5B). Treatment of POSTN in control animals that were not subjected to HI (Sham + POSTN) did not enhance performance over basal levels (Sham + PBS), indicating that the effects of POSTN on cognitive function were specific to the conditions produced by HI. In the probe trials, animals in the HI + PBS group spent less time in the target quadrant (Fig 5C) and engaged in fewer platform crossings (Fig 5D) as compared to control animals. Treatment of POSTN in HI animals restored the number of platform crossings and the time spent in the target quadrant to values comparable to those of control animals.

## Discussion

Periostin is characterized as a matricellular protein with known functions in different physiological and pathological conditions [29], especially in tissue remodeling as a response to insult/injury. Our present study demonstrated that POSTN mRNA was stably expressed in NSCs, and deficiency of POSTN depressed the proliferation and differentiation of NSCs. The proliferation and differentiation of cultured NSCs were stimulated after POSTN treatment. POSTN promoted the neurogenesis and function recovery in HI brain injury. Taken together, these findings indicate that POSTN plays an important role in brain development and brain injury remodeling.

Emerging evidences have shown that during embryogenesis and in the neonate, expressions of POSTN isoforms show a specific temporal and spatial pattern, which suggests different functions in tissue development and remodeling [29]. Zhu *et al*. found that the effect of POSTN on bone formation and remodeling is through an increase in osteoblast proliferation, differentiation, adhesion, and survival [30]. Furthermore, POSTN has been shown to be expressed at basal levels in healthy human skin but localized to the extracellular compartment during tissue remodeling involved in wound repair [31]. POSTN plays a key role in cardiovascular development, however, the expression of POSTN can be rapidly increased in response to injury [32–35]. The previous studies have shown that POSTN has an effect on promoting non-neuronal cell genesis in development and pathology [30, 36, 37]. However, our present study suggested that POSTN also expresses in NSCs and plays important roles in brain development and remodeling.

POSTN also has a neuroprotective role *in vitro* and *in vivo* during cerebral ischemia [38]. Shimamura *et al*. reported that POSTN expressed in various cells, such as reactive astrocytes/microglia, fibroblasts, and neuronal progenitor cells, and might be associated with pathophysiology in post-ischemic inflammation and neurogenesis [39]. However, there are no reports that suggest a role of POSTN in the neonatal hypoxic-ischemic rodent brains, especially a potential role in stimulating proliferation of NSCs and promoting neurogenesis following injury.

In the present study, it was observed that POSTN mRNA levels were increased upon NSC differentiation. These data suggest that even under non-pathological conditions, endogenous POSTN is associated with NSC differentiation. Indeed, POSTN treatment significantly enhanced NSC differentiation into neurons and astrocytes *in vitro* (Fig 2). Consistent with results from previous studies [40, 41], following hypoxic-ischemic injury, POSTN treatment in neonatal rat brains also enhanced the proliferation and differentiation of NSCs in two neurogenic regions, the SVZ (Fig 3) and the SGZ (Fig 4). HI in itself increases NSC self-renewal, differentiation and maturation [42]. However, endogenous neurogenesis following HI injury is ineffective on repairing damage; studies have shown that the capacity for neuronal replacement in the adult brain is very low, with only an estimated 0.2% to 10% of lost neurons being replaced [43, 44]. The results described herein suggest that POSTN has an effect on stimulating neuron production following HI beyond basal levels.

Factors that promote NSC proliferation and differentiation may be useful as potential therapeutic tools for treating brain injury [45, 46]. In the present study, POSTN significantly enhanced NSC proliferation and differentiation when applied exogenously; moreover, Morris water maze test showed that POSTN-treated animals had a significant improvement in spatial learning and memory, which were impaired by HI, compared to untreated control animals. These results indicate that POSTN may reverse cognitive deficits resulting from HI injury by stimulating NSC self-renewal and neuronal and glial cell differentiation.

Periostin is thought to play a critical role in cell apoptosis. Recent study has found that periostin silencing significantly increased the number of apoptotic colon cancer cells through supressing the PI3K/Akt/survivin pathway [47]. Aukkarasongsup *et al*. [48] also demonstrated that the percentage of apoptotic periodontal ligament cells was markedly decreased under hypoxia after treatment with recombinant human POSTN via TGF-β signaling. However, other study has shown that POSTN deficiency decreased the hypoxia-stimulated proliferation, but had no effect on cell apoptosis [49]. In our study, we tested the anti-apoptosis effect of POSTN on neurons and found that POSTN protected neurons from apoptosis under hypoxia conditions (data not shown). The anti-apoptosis effect of POSTN on NSCs under hypoxia and the related molecular mechanism need the further study.

## Conclusions

In summary, POSTN treatment significantly enhanced NSC proliferation and stimulated the production of neurons and astrocytes both *in vitro* and *in vivo*. Furthermore, POSTN improved cognitive functions that were compromised by HI-induced brain injury. Additional studies need to be undertaken in order to examine the mechanisms underlying POSTN-induced stimulation of neurogenesis for these findings to be useful in the development of clinical approaches for the treatment of perinatal HI, and improving the prognosis of HI encephalopathy and other types of brain injury.

## References

[1] Wachtel EV, Hendricks-Munoz KD. Current management of the infant who presents with neonatal encephalopathy. Curr Probl Pediatr Adolesc Health Care. 2011;41: 132–153. 10.1016/j.cppeds.2010.12.002 21458747

[2] Ferriero DM. Neonatal brain injury. N Engl J Med. 2004;351: 1985–1995. 15525724 10.1056/NEJMra041996

[3] Gonzalez FF, Ferriero DM. Therapeutics for neonatal brain injury. Pharmacol Ther. 2008;120: 43–53. 10.1016/j.pharmthera.2008.07.003 18718848

[4] Pimentel-Coelho PM, Mendez-Otero R. Cell therapy for neonatal hypoxic-ischemic encephalopathy. Stem Cells Dev. 2010;19: 299–310. 10.1089/scd.2009.0403 19916801

[5] Ming GL, Song H. Adult neurogenesis in the mammalian brain: significant answers and significant questions. Neuron. 2011;70: 687–702. 10.1016/j.neuron.2011.05.001 21609825 PMC3106107

[6] Weiss S, Reynolds BA, Vescovi AL, Morshead C, Craig CG, van der Kooy D. Is there a neural stem cell in the mammalian forebrain? Trends Neurosci. 1996;19: 387–393. 8873356 10.1016/s0166-2236(96)10035-7

[7] Hayashi T, Iwai M, Ikeda T, Jin G, Dequchi K, Nagotani S, et al Neural precursor cells division and migration in neonatal rat brain after ischemic/hypoxic injury. Brain Res. 2005;1038: 41–49. 15748871 10.1016/j.brainres.2004.12.048

[8] Ikeda T, Iwai M, Hayashi T, Nagano I, Shogi M, Ikenoue T, et al Limited differentiation to neurons and astroglia from neural stem cells in the cortex and striatum after ischemia/hypoxia in the neonatal rat brain. Am J Obstet Gynecol. 2005;193: 849–856. 16150285 10.1016/j.ajog.2005.01.029

[9] Iwai M, Ikeda T, Hayashi T, Sato K, Nagata T, Nagano I, et al Temporal profile of neural stem cell proliferation in the subventricular zone after ischemia/hypoxia in the neonatal rat brain. Neurol Res. 2006;28: 461–468. 16759450 10.1179/016164105X49283

[10] Ikeda T. Stem cells and neonatal brain injury. Cell Tissue Res. 2008;331: 263–269. 18040721 10.1007/s00441-007-0546-8

[11] Conway SJ, Izuhara K, Kudo Y, Litvin J, Markwald R, Ouyang G, et al The role of periostin in tissue remodeling across health and disease. Cell Mol Life Sci. 2014;71: 1279–1288. 10.1007/s00018-013-1494-y 24146092 PMC3949008

[12] Shih CH, Lacagnina M, Leuer-Bisciotti K, Pröschel C. Astroglial-derived periostin promotes axonal regeneration after spinal cord injury. J Neurosci. 2014;34: 2438–2443. 10.1523/JNEUROSCI.2947-13.2014 24523534 PMC6802751

[13] Zhu S, Barbe MF, Amin N, Rani S, Popoff SN, Safadi FF, et al Immunolocalization of Periostin-like factor and Periostin during embryogenesis. J Histochem Cytochem. 2008;56: 329–345. 18040074 10.1369/jhc.7A7321.2007 PMC2326108

[14] Oka T, Xu J, Kaiser RA, Melendez J, Hambleton M, Sargent MA, et al Genetic manipulation of periostin expression reveals a role in cardiac hypertrophy and ventricular remodeling. Circ Res. 2007;101: 313–321. 17569887 10.1161/CIRCRESAHA.107.149047 PMC2680305

[15] Zhu S, Barbe MF, Liu C, Hadjiargyrou M, Popoff SN, Rani S, et al Periostin-like-factor in osteogenesis. J Cell Physiol. 2009;218: 584–592. 10.1002/jcp.21633 19006175

[16] Nishiyama T, Kii I, Kashima TG, Kikuchi Y, Ohazama A, Shimazaki M, et al Delayed re-epithelialization in periostin-deficient mice during cutaneous wound healing. PLoS One. 2011;6: e18410 10.1371/journal.pone.0018410 21490918 PMC3072397

[17] Kuhn B, del Monte F, Hajjar RJ, Chang YS, Lebeche D, Arab S, et al Periostin induces proliferation of differentiated cardiomyocytes and promotes cardiac repair. Nat Med. 2007;13: 962–969. 17632525 10.1038/nm1619

[18] Dorn GN. Periostin and myocardial repair, regeneration, and recovery. N Engl J Med. 2007;357: 1552–1554. 17928607 10.1056/NEJMcibr074816

[19] Takeshita S, Kikuno R, Tezuka K, Amann E. Osteoblast-specific factor 2: cloning of a putative bone adhesion protein with homology with the insect protein fasciclin I. Biochem J. 1993;294: 271–278. 8363580 10.1042/bj2940271 PMC1134594

[20] Shimamura M, Taniyama Y, Katsuragi N, Koibuchi N, Kyutoku M, Sato N, et al Role of central nervous system periostin in cerebral ischemia. Stroke. 2012;43: 1108–1114. 10.1161/STROKEAHA.111.636662 22308244

[21] Taupin P. BrdU immunohistochemistry for studying adult neurogenesis: paradigms, pitfalls, limitations, and validation. Brain Res Rev. 2007;53: 198–214. 17020783 10.1016/j.brainresrev.2006.08.002

[22] Sheldon RA, Chuai J, Ferriero DM. A rat model for hypoxic-ischemic brain damage in very premature infants. Biol Neonate. 1996;69: 327–341. 8790911 10.1159/000244327

[23] Vannucci RC, Connor JR, Mauger DT, Palmer C, Smith MB, Towfighi J, et al Rat model of perinatal hypoxic-ischemic brain damage. J Neurosci Res. 1999;55: 158–163. 9972818 10.1002/(SICI)1097-4547(19990115)55:2<158::AID-JNR3>3.0.CO;2-1

[24] Huang Z, Liu J, Cheung PY, Chen C. Long-term cognitive impairment and myelination deficiency in a rat model of perinatal hypoxic-ischemic brain injury. Brain Res. 2009;1301: 100–109. 10.1016/j.brainres.2009.09.006 19747899

[25] Sun J, Sha B, Zhou W, Yang Y. VEGF-mediated angiogenesis stimulates neural stem cell proliferation and differentiation in the premature brain. Biochem Biophys Res Commun. 2010;394: 146–152. 10.1016/j.bbrc.2010.02.132 20188072

[26] Yan H, Zhou W, Wei L, Zhong F, Yang Y. Proteomic analysis of astrocytic secretion that regulates neurogenesis using quantitative amine-specific isobaric tagging. Biochem Biophys Res Commun. 2010;391: 1187–1191. 10.1016/j.bbrc.2009.12.015 20005204

[27] Vorhees CV, Williams MT. Morris water maze: procedures for assessing spatial and related forms of learning and memory. Nat Protoc. 2006;1: 848–858. 17406317 10.1038/nprot.2006.116 PMC2895266

[28] Xiong M, Zhang T, Zhang LM, Lu SD, Huang YL, Sun FY. Caspase inhibition attenuates accumulation of beta-amyloid by reducing beta-secretase production and activity in rat brains after stroke. Neurobiol Dis. 2008;32: 433–441. 10.1016/j.nbd.2008.08.007 18805488

[29] Conway SJ, Izuhara K, Kudo Y, Litvin J, Markwald R, Ouyang G, et al The role of periostin in tissue remodeling across health and disease. Cell Mol Life Sci. 2014;71: 1279–1288. 10.1007/s00018-013-1494-y 24146092 PMC3949008

[30] Zhu S, Barbe MF, Liu C, Hadjiargyrou M, Popoff SN, Rani S, et al Periostin-like-factor in osteogenesis. J Cell Physiol. 2009;218: 584–592. 10.1002/jcp.21633 19006175

[31] Jackson-Boeters L, Wen W, Hamilton DW. Periostin localizes to cells in normal skin, but is associated with the extracellular matrix during wound repair. J Cell Commun Signal. 2009;3: 125–133. 10.1007/s12079-009-0057-3 19543815 PMC2721086

[32] Snider P, Hinton RB, Moreno-Rodriguez RA, Wang J, Rogers R, Lindsley A, et al Periostin is required for maturation and extracellular matrix stabilization of noncardiomyocyte lineages of the heart. Circ Res. 2008;102: 752–760. 10.1161/CIRCRESAHA.107.159517 18296617 PMC2754697

[33] Conway SJ, Molkentin JD. Periostin as a heterofunctional regulator of cardiac development and disease. Curr Genomics. 2008;9: 548–555. 10.2174/138920208786847917 19516962 PMC2694556

[34] Zhou B, von Gise A, Ma Q, Hu YW, Pu WT. Genetic fate mapping demonstrates contribution of epicardium-derived cells to the annulus fibrosis of the mammalian heart. Dev Biol. 2010;338: 251–261. 10.1016/j.ydbio.2009.12.007 20025864 PMC2815244

[35] Stansfield WE, Andersen NM, Tang RH, Selzman CH. Periostin is a novel factor in cardiac remodeling after experimental and clinical unloading of the failing heart. Ann Thorac Surg. 2009;88: 1916–1921. 10.1016/j.athoracsur.2009.07.038 19932262 PMC3686640

[36] Ishikawa K, Yoshida S, Nakao S, Nakama T, Kita T, Asato R, et al Periostin promotes the generation of fibrous membranes in proliferative vitreoretinopathy. FASEB J. 2014;28: 131–142. 10.1096/fj.13-229740 24022401

[37] Padial-Molina M, Volk SL, Rios HF. Periostin increases migration and proliferation of human periodontal ligament fibroblasts challenged by tumor necrosis factor-alpha and Porphyromonas gingivalis lipopolysaccharides. J Periodontal Res. 2014;49: 405–414. 10.1111/jre.12120 23919658

[38] Shimamura M, Taniyama Y, Katsuragi N, Koibuchi N, Kyutoku M, Sato N, et al Role of central nervous system periostin in cerebral ischemia. Stroke. 2012;43: 1108–1114. 10.1161/STROKEAHA.111.636662 22308244

[39] Shimamura M, Taniyama Y, Nakagami H, Katsuragi N, Wakayama K, Koriyama H, et al Long-term expression of periostin during the chronic stage of ischemic stroke in mice. Hypertens Res. 2014;37: 494–499. 10.1038/hr.2014.36 24646642

[40] Ohira K. Injury-induced neurogenesis in the mammalian forebrain. Cell Mol Life Sci. 2011;68: 1645–1656. 10.1007/s00018-010-0552-y 21042833 PMC11115059

[41] Dizon M, Szele F, Kessler JA. Hypoxia-ischemia induces an endogenous reparative response by local neural progenitors in the postnatal mouse telencephalon. Dev Neurosci. 2010;32: 173–183. 10.1159/000313468 20616554 PMC2941849

[42] De Filippis L, Delia D. Hypoxia in the regulation of neural stem cells. Cell Mol Life Sci. 2011;68: 2831–2844. 10.1007/s00018-011-0723-5 21584807 PMC11115125

[43] Arvidsson A, Collin T, Kirik D, Kokaia Z, Lindvall O. Neuronal replacement from endogenous precursors in the adult brain after stroke. Nat Med. 2002;8: 963–970. 12161747 10.1038/nm747

[44] Nakatomi H, Kuriu T, Okabe S, Yamamoto S, Hatano O, Kawahara N, et al Regeneration of hippocampal pyramidal neurons after ischemic brain injury by recruitment of endogenous neural progenitors. Cell. 2002;110: 429–441. 12202033 10.1016/s0092-8674(02)00862-0

[45] Alvarez-Buylla A, Lim DA. For the long run: maintaining germinal niches in the adult brain. Neuron. 2004;41: 683–686. 15003168 10.1016/s0896-6273(04)00111-4

[46] Lim DA, Tramontin AD, Trevejo JM, Herrera DG, García-Verdugo JM, Alvarez-Buylla A. Noggin antagonizes BMP signaling to create a niche for adult neurogenesis. Neuron. 2000;28: 713–726. 11163261 10.1016/s0896-6273(00)00148-3

[47] Xiao ZM, Wang XY, Wang AM. Periostin induces chemoresistance in colon cancer cells through activation of the PI3K/Akt/survivin pathway. Biotechnol Appl Biochem. 2013 12 24 10.1002/bab.1193.s 24372557

[48] Aukkarasongsup P, Haruyama N, Matsumoto T, Shiga M, Moriyama K. Periostin inhibits hypoxia-induced apoptosis in human periodontal ligament cells via TGF-β signaling. Biochem Biophys Res Commun. 2013;441: 126–132. 10.1016/j.bbrc.2013.10.027 24129188

[49] Zhang Z. Increased periostin expression affects the proliferation, collagen synthesis, migration and invasion of keloid fibroblasts under hypoxic conditions. Int J Mol Med. 2014;34: 253–261. 10.3892/ijmm.2014.1760 24788198

